# Re: Transition to adulthood with a bladder augmentation: histopathologic concerns

**DOI:** 10.1590/S1677-5538.IBJU.2018.0027

**Published:** 2018

**Authors:** Ines Mendes Pina, Ahmed M. Omar, Rauf N. Khadr, Michael S. Floyd

**Affiliations:** 1 Department of Reconstructive Urology, St Helens & Knowsley Hospital NHS Trust, Whiston Hospital, Liverpool, United Kingdom, UK; 2 Northwest Regional Spinal Cord Injury Unit, Southport & Ormskirk NHS Foundation Trust, Town Lane, Kew, Merseyside, United Kingdom, UK

*To the editor,*

We read with interest the recent paper by Mammadov et al. examining the concerns regarding histological changes in adult neurogenic patients who have undergone bladder augmentation with bowel interposition as an adolescent or in childhood (1).

The authors report a small study involving 20 patients who underwent selective anatomic bladder biopsies following augmentation for either neurogenic bladder, exstrophy or bladder neck trauma.

Two patients underwent open bladder biopsy as a simultaneous stone extraction procedure was planned. Neuropathic patients with a reconstructed or ablated urethra pose a challenge for the Urologist as they do not have dependant bladder drainage (2). Specific to the neurogenic patient with bladder calculi the authors should acknowledge that mitrofanoff cystolitholopaxy and concomitant bladder biopsy has been reported using a minimally invasive, hybrid technique thus avoiding the morbidity of open surgery (3).

Mammadov et al. reported no malignant histology in the study but did detect 2 cases of squamous metaplasia and 1 case of intestinal metaplasia (1). One case of squamous metaplasia had a history of bladder stones similar to the case reported by Floyd Jr et al. (3).

In 2011, Higuchi at al examined 250 surveillance cystoscopies and although 4 lesions were identified, none were malignant leading the authors to conclude that annual surveillance cystoscopy was not cost effective (4). A separate publication by Higuchi at al also commented on immunosuppression as an independent risk factor for neoplastic development in bladder augmentation patients (5) and this has not been addressed by Mammadov et al.

The authors conclude by stating that surveillance cystoscopy in augmented patients less than 5 years post operatively is now limited to those with symptoms. This is a very pertinent clinical point. Hamid et al. detected no malignancy in a series of 92 augmented and substituted patients undergoing surveillance cystoscopy but detected higher rates of chronic inflammation in the augmented group (6). Furthermore, they concluded by stating that surveillance cystoscopy was not merited in patients less than 15 years post operatively but advised that investigations should be prompted by development of appropriate symptoms in these patients (6).
